# Do Work Engagement and Transformational Leadership Facilitate Knowledge Sharing? A Perspective of Conservation of Resources Theory

**DOI:** 10.3390/ijerph17072615

**Published:** 2020-04-10

**Authors:** Wei-Li Wu, Yi-Chih Lee

**Affiliations:** Department of International Business, Chien Hsin University of Science and Technology, Taoyuan 32097, Taiwan; leeyc@uch.edu.tw

**Keywords:** knowledge sharing, transformational leadership, work engagement, intrinsic motivation, COR theory, multilevel research

## Abstract

Based on the perspective of conservation of resources (COR) theory, this study adopts a multilevel approach to examine the influences of employees’ personal resources (i.e., work engagement and intrinsic motivation) and external resources (i.e., transformational leadership) on knowledge sharing. This study conducts a survey to explore the interrelationships among transformational leadership, work engagement, intrinsic motivation, and knowledge sharing. The sample includes 33 healthcare work groups consisting of 214 group members. The results show that an individual’s personal and external resources are positive and benefit the promotion of knowledge sharing. As for personal resources, work engagement has a positive impact on knowledge sharing by increasing intrinsic motivation. Regarding external resources, transformational leadership acts as a facilitator for knowledge sharing. Specifically, the conditional indirect effects of work engagement on knowledge sharing through intrinsic motivation are more positive under high levels of transformational leadership, rather than low levels of transformational leadership. Based on the COR theory, this is the first study to argue that knowledge sharing could be considered as an active activity and that individuals could be eager to perform knowledge sharing when they possess significant personal and external resources. The results of this study provide new insights into knowledge sharing.

## 1. Introduction

Knowledge sharing is the foundation of successful knowledge management [1,2]. For organizations, successful knowledge sharing helps organizations achieve competitive advantages and sustainable operations. Although employees’ knowledge sharing obviously benefits their organization, the current research on knowledge sharing somehow hypothesizes that knowledge sharing might be bad for employees because once employees have shared their knowledge with colleagues, they lose the monopoly of knowledge. However, this study considers that when employees perform knowledge sharing at work, it might be also good for them. For example, when employees engage in knowledge-sharing behaviors, they can gain more self-affirmation and expand their interpersonal relationships with colleagues. These positive psychological perceptions are all important factors that constitute the psychological well-being of employees [3] and help them achieve a better level of psychological health. In fact, successful knowledge sharing might benefit both organizations’ survival and employees’ well-being. As a result, it is important to know the ways to promote employees’ knowledge-sharing behavior.

Previous studies have exerted much effort to understand the determinants of employee knowledge sharing. Employees might share their knowledge because of environment factors, individual characteristics and/or motivational factors [4]. Social exchange theory [5] is the core theoretical perspective used by previous studies. Based on the social exchange theory, previous studies have shown that employee tend to perform knowledge sharing when they perceive higher levels of organizational procedural justice [6,7], organizational support [8], trust [9] or positive leadership [10]. In addition, if companies offer a positive and friendly working environment for their employees, it could promote employees’ knowledge sharing. Basically, managers could make a better knowledge-sharing environment through a human resource management system [11,12], positive organization climate [13] or job design [14]. Furthermore, in recent years, scholars have started to discuss factors that might decrease employees’ level of knowledge sharing, such as abusive supervision [15,16]. In sum, previous studies have provided abundant findings regarding the antecedents of knowledge sharing.

However, three shortcomings still seem to exist in the extant literature. First, previous studies usually hypothesize that knowledge sharing is not a kind of activity that employees tend to perform actively. Employees might face a social dilemma when they think about sharing knowledge [17,18] because once employees share their knowledge with others, their knowledge might become a public good. According to social exchange theory, individuals are not prone to engage in knowledge sharing unless they perceive gaining positive benefits from organizations such as organizational support or positive leadership [8,10]. Thus, knowledge sharing is often considered as a passive action by previous studies. Second, most of the knowledge sharing research supposes that, since knowledge is power, engaging in knowledge sharing is likely to result in a loss of power [19,20]. Third, although an individual’s performance of knowledge sharing is supposed to be influenced by multi-level factors simultaneously, knowledge sharing research adopting a multilevel perspective is still in its initial stages [10]. 

In this study, we consider knowledge sharing from a different angle. If knowledge is power, then this means that knowledge is an important resource. Therefore, people ought to be keen to accumulate knowledge [15]. In fact, knowledge sharing is one means of accumulating knowledge; during the process of knowledge sharing, individuals have the chance to engage in mutual learning [21] and thereby gain more knowledge. As a result, this study posits that knowledge sharing can also be considered an active action and a means of gaining resources. More specifically, individuals might be intrinsically motivated to actively engage in knowledge sharing to accumulate an important resource, namely knowledge. However, we know little about the process and determinants that cause individuals to actively pursue knowledge sharing. 

This study applies the conservation of resources (COR) theory to discuss why individuals actively perform knowledge sharing. The conservation of resources theory is a motivational theory according to which individuals strive to obtain, retain, foster, and protect valued resources [22,23]. The conservation of resources theory has been used to discuss resource loss over a long period of time [24,25]. Researchers in recent years have begun to consider it as an important perspective for understanding how people gain resources [26]. There are three reasons why this study uses the COR theory as the main theoretical lens. First, knowledge is an important resource and the COR theory is the primary theory used to discuss how individuals deal with resources [27]. Second, the COR theory proposes the “gain spiral of resources” concept [28], which is suitable for discussing how individuals use current resources to promote knowledge sharing. Third, the COR theory is a well-developed theory [27]. In applying this theoretical lens to knowledge sharing research, this study is able to obtain a new and insightful perspective on exploring the important determinants of knowledge sharing [15,16].

According to the COR theory, people will invest resources in order to obtain resources. People who have abundant resources are usually in a better position to garner more resources [29,30]. Knowledge is an important resource and knowledge sharing is an important way of obtaining resources. Therefore, when employees have more resources, it is easier for them to become involved in knowledge sharing. This study uses work engagement to represent an employee’s personal resources. Work engagement is defined as an active, positive, work-related state of mind that is characterized by vigor, dedication, and absorption [31]. Engaged employees are full of energy and have abundant resources [32,33,34]. According to the COR theory, engaged employees have more resources to invest in obtaining further resources and so engage in more knowledge sharing compared to disengaged employees. Furthermore, this study also explores the relationship between work engagement and knowledge sharing. Engaged employees highly value and love their work; they therefore tend to be intrinsically motivated to perform knowledge sharing. As a result, this study argues that work engagement is indirectly related to knowledge sharing via intrinsic motivation. 

**Figure 1:**
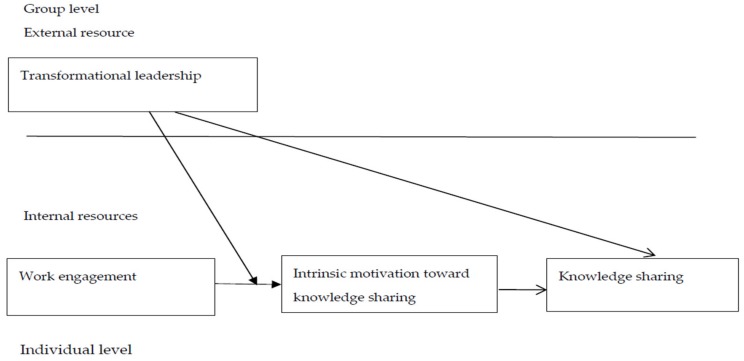
Research framework.

According to the COR theory, external resources are an important source for people [35]; thus, this study also considers the influences of employees’ external resources on their knowledge sharing performance. In this study, employees’ external resources are represented by transformational leadership, a determinant at the group level. Knowledge sharing usually occurs in a group context. Within a group, leadership has a significant impact on members’ behavior, including knowledge sharing. Transformational leadership has been proven to be an effective and positive leadership style [36,37,38,39]. More importantly, transformational leadership has been shown to have a positive influence on increasing employees’ psychological resources, such as self-efficacy [40,41] or positive mood [42]. When employees have a higher level of psychological resources, it leads to a higher level of knowledge sharing [10]. Thus, group leaders’ transformational leadership could be considered as an external supporting resource for promoting employees’ knowledge sharing. Furthermore, the COR theory also argues that different resources aggregate into a resource pool [43], and therefore have a conjunctive influence. Accordingly, this study argues that work engagement and transformational leadership might also have a conjunctive influence on intrinsic motivation toward knowledge sharing. Previous studies have shown that transformational leaders can make their followers feel a higher level of meaningfulness in their work [44,45]. Therefore, when engaged employees work under transformational leaders, they might be more eager to participate in knowledge sharing through their own autonomous decision. Transformational leaders can also make followers feel a stronger sense of social support [46], which helps engaged employees feel safer to make their own decisions, such as knowledge sharing. In sum, we suppose that transformational leadership would strengthen the relationship between work engagement and intrinsic motivation toward knowledge sharing. The theoretical framework of this study is shown in Figure 1. 

We hope the results of this study could make three theoretical contributions. First, this study hypothesizes that knowledge sharing is an important way of obtaining knowledge. Therefore, employees might be actively motivated to pursue knowledge sharing once they have abundant resources. From the perspective of the COR theory, we can explore the determinants of knowledge sharing in a new way. Second, this study considers both work engagement and transformational leadership as important and positive resources; it responds to the call of scholars of positive organizational behavior for further study to emphasize the positive side of people as well as the impact of positive leadership. Finally, by combining the COR theory and knowledge sharing research, this study further extends the theoretical application of the COR theory.

### 1.1. Theoretical Development

#### 1.1.1. Knowledge Sharing and Work Engagement

Knowledge sharing is a very important part of knowledge management. In the process of knowledge sharing, knowledge donation and collection occur [21,47]. Knowledge donation means that knowledge possessors provide their knowledge to others. Knowledge collection refers to the fact that knowledge receivers acquire new knowledge from others. As a result, knowledge sharing provides a way for employees to teach and learn, and benefits employees by increasing their personal knowledge. According to the COR theory [22,23], because knowledge is an important resource, employees should have higher levels of motivation to accumulate knowledge. Since the process of knowledge sharing provides employees with a chance for mutual learning, it is a good way to accumulate knowledge. As a result, knowledge sharing is not just about sharing resources, but also about gaining resources.

Work engagement refers to an active, positive, fulfilling, work-related state of mind characterized by vigor, dedication, and absorption [31,48]. In the previous studies, work engagement has been proven to be positively related to desirable outcomes, such as innovative behavior [49] or organizational citizenship behavior [50]. According to COR, individuals with more resources are more likely to invest in future resources [29,30]. This study infers that work engagement positively influences employees’ knowledge sharing. Gorgievski and Hobfoll argue that work engagement is the end state of a long-term process of gaining resources [51]. In other words, engaged employees will have abundant resources to invest in their jobs [33] and subsequently obtain more resources. Similarly, this study hypothesizes that engaged employees have abundant resources to bring to the process of knowledge sharing in order to obtain new knowledge. 

Regarding donating knowledge, since engaged employees are deeply involved in their work, they should have more work-related knowledge to provide. More importantly, because engaged employees value their work quite highly, this study argues that they tend to be more willing to provide their current knowledge in order to exchange future knowledge. Consistent with the principle of the COR theory that people invest resources to gain resources [29,30], this study infers that engaged employees will be more positive regarding donating knowledge compared to disengaged employees. As for knowledge collecting, because engaged employees are more energetic and dedicated to their work, they will acquire more knowledge in the process of knowledge sharing. Learning and collecting knowledge could be a challenging task for employees, since the new knowledge might not be in the same field as the one they are familiar with. Since engaged employees are more energetic [51], they have more resources to cope with the difficulties faced during knowledge collecting. As a result, based on the above discussion, this study expects that engaged employees are better at both knowledge donating and collecting, and thus experience better knowledge sharing.

**Hypothesis** **1.** *The greater the degree of work engagement the employees has, the more knowledge sharing the employees will perform.*

#### 1.1.2. The Mediating Influence of Intrinsic Motivation

Since engaged employees devote themselves to their work and could be involved in knowledge sharing as one way to gain more work knowledge, it is reasonable to hypothesize that engaged employees will engage in knowledge sharing primarily based on their own interests and enjoyment. In other words, engaged employees might have a higher level of intrinsic motivation toward knowledge sharing. As a result, intrinsic motivation toward knowledge sharing might be an important mediator in the relationship between work engagement and knowledge sharing. There might be a work engagement–intrinsic motivation–knowledge sharing causal chain relationship. 

In order to have a clearer explanation, we combined some insights gained from the self-determination theory (SDT) [52,53] with the COR theory in order to theorize this causal chain relationship. Applying insights from SDT to elaborate the COR model is also suggested by, and used in, previous studies [54,55]. Consistent with the definition of Foss et al. (2009) [14], intrinsic motivation in this study refers to intrinsic motivation toward knowledge sharing. According to SDT [52,53], intrinsic motivation is a desire to engage in an activity because of the pleasure and interest derived from the activity itself. People will have a higher level of intrinsic motivation toward a certain activity when the psychological needs for autonomy, competence and relatedness are satisfied by engaging in the activity. In addition, since intrinsic motivation energizes individuals, it is also a personal resource [55]. 

This study hypothesizes that engaged employees will have a higher level of intrinsic motivation than disengaged employees. The main reason behind this hypothesis is that engaged employees are more likely to have satisfied psychological needs (i.e., autonomy and competence) when it comes to knowledge sharing compared to disengaged ones. First, regarding the need for autonomy, this study argues that engaged employees will experience more satisfaction in relation to the enhancement of their autonomy than disengaged employees. Since engaged employees love their work [31,48], they will autonomously try to make their work better or establish a better work environment [56]. As we described above, knowledge sharing could be an important means to acquire new work-related knowledge; thus, engaged employees should be autonomously motivated to engage in knowledge sharing. Then, engaged employees should have higher degrees of satisfaction of their autonomy than disengaged ones when it comes to knowledge sharing. Second, in terms of the need for a sense of competence, this study argues that engaged employees feel more competent than disengaged employees do. Since engaged employees usually experience positive emotions, they are able to create more resources in their daily work-related tasks [56,57]. It is therefore reasonable to hypothesize that engaged employees have a better ability to mobilize and increase resources to perform knowledge sharing, or to deal with the difficulties resulting from knowledge sharing. As a result, engaged employees would show more confidence and feel competent in their knowledge sharing. Based on the above descriptions, engaged employees should experience and feel more satisfaction in relation to a sense of autonomy and competence when it comes to knowledge compared to disengaged employees. According to SDT [52,53], when psychological needs (autonomy and competence) are more fulfilled, this can lead to a higher level of intrinsic motivation. Therefore, work engagement should be positively related to intrinsic motivation toward knowledge sharing.

**Hypothesis** **2.** *The greater the degree of work engagement the employees has, the more the employee will tend to be intrinsically motivated to share knowledge.*

According to the COR theory, intrinsic motivation is an important personal resource because it can help an individual attain his or her goals [23,54]. Intrinsic motivation toward knowledge sharing implies that individuals are actively motivated to engage in knowledge sharing, and find it to be interesting, enjoyable and joyful. Regarding the relationship between intrinsic motivation and knowledge sharing, previous studies clearly point out that intrinsically motivated employees are more likely to engage in knowledge sharing [14,58,59,60]. In contrast, those employees with low intrinsic motivation might need to be pushed to share knowledge [61]. Therefore, consistent with previous studies, intrinsic motivation toward knowledge sharing will have a positive influence on knowledge sharing. Combining this inference with Hypothesis 2, we argue that engaged employees are positively related to intrinsic motivation, which then leads to greater knowledge sharing.

**Hypothesis** **3.** *Intrinsic motivation will mediate the relationship between work engagement and knowledge sharing.*

#### 1.1.3. Transformational Leadership

Transformational leadership is a positive leadership style. Previous studies have shown that transformational leadership can encourage and promote positive outcomes in followers [37,38,39,62]. Transformational leadership is composed of four dimensions: idealized influence, inspirational motivation, intellectual stimulation, and individualized consideration [63,64]. According to the COR theory, job resources are an important external resource [29,30]. Of these job resources, positive leadership represents one kind of important resource. In this study, transformational leadership within a group represents an external resource for employees.

Previous studies have shown that transformational leadership can help followers to develop two kinds of personal resources, namely self-efficacy [40] and positive affect [42]. Both self-efficacy and positive affect are important personal and positive psychological resources [35], and can help to promote knowledge sharing [10]. When employees have higher levels of positive psychological resources, they will be more likely to engage in knowledge donating because they are more positive and optimistic in their view of donating knowledge. Moreover, they will have more positive thoughts and actions when involved in learning, and will thus exhibit a higher level of knowledge collection. In other words, within a group, transformational leaders promote employees’ positive psychological resources and this leads to greater knowledge sharing behavior. Therefore, we argue that transformational leadership within a group is positively related to employee knowledge sharing.

**Hypothesis** **4.** *The greater the degree of transformational leadership within a group, the more employees will perform knowledge sharing.*

According to the COR theory [43], an individual’s resources form a resource pool and then exhibit an aggregating influence. In this study, we discuss two different kinds of resource for an employee, namely work engagement and transformational leadership, which represent a personal resource and an external resource, respectively. Based on the resource pool concept of the COR theory, work engagement and transformational leadership might have a conjunctive influence on the process of knowledge sharing. More specifically, this study argues that transformational leadership might enhance the relationship between work engagement and intrinsic motivation for two reasons. First, transformational leaders can make their followers feel more supported [46]; with this perception, engaged employees could feel safer making their own decisions, such as engaging in knowledge sharing. Therefore, engaged employees will have a higher level of automatic satisfaction when engaging in knowledge sharing under the lead of transformational leaders than under non-transformational leaders. Second, transformational leaders tend to more positively motivate and inspire their followers [63,64]; therefore, their followers will exhibit more confidence in the workplace. Previous studies have shown that transformational leadership leads to positive psychological states in followers [38,65,66]. Therefore, under the mentoring and coaching of transformational leaders, engaged employees will have more confidence to engage in knowledge sharing than under non-transformational leaders because they experience more positive psychological states which further strengthen their confidence in knowledge sharing. As a result, transformational leadership should positively moderate the relationship between work engagement and intrinsic motivation.

**Hypothesis** **5.** *Transformational leadership will moderate the relationship between work engagement and intrinsic motivation. When transformational leadership is high, the positive relationship between work engagement and intrinsic motivation is increased.*

Furthermore, since transformational leadership strengthens the impact of work engagement on intrinsic motivation, it could also change the indirect effect of work engagement on knowledge sharing via intrinsic motivation. Integrating Hypothesis 3 and Hypothesis 5, we therefore argue that the mediating effect of intrinsic motivation on the relationship between work engagement and knowledge sharing will vary as a function of transformational leadership.

**Hypothesis** **6.** *The conditional indirect effect of work engagement on knowledge sharing via intrinsic motivation will be stronger when transformational leadership is high than when transformational leadership is low.*

## 2. Materials and Methods 

### 2.1. Sample 

Since this study deals with an issue involving both the individual and group levels, we collected the data by the unit of the work group. In addition, previous studies have pointed out that knowledge sharing is an important activity for healthcare workers [67,68]; thus, we chose healthcare work groups as the targeted sample. Since group questionnaires are more difficult to collect, this study used purposive sampling for the survey in Taiwan. To ensure the usability of the group questionnaires, and by referring to the collection approaches of group questionnaires in previous studies, a valid group questionnaire must consist of the leader questionnaire and at least three group members [15,69].

The authors contacted the hospitals via telephone to ask whether they were willing to participate; a medical center and a regional hospital agreed to join this research. The medical institutions are divided into four levels in Taiwan: “clinic”, “district hospital”, “regional hospital” and “medical center”. The higher the medical level, the larger the scale, the more relevant medical equipment and medical staff, the more specialized. There is one regional hospital per 400,000 people in Taiwan, with more than 300 beds. There is one medical center per 2 million people with more than 500 beds [70].

Due to the suggestion from the hospitals, as medical doctors are highly professional and their workload is extremely high, this study does not include the sample of medical doctors. Questionnaires were sent to the hospitals via delivery or in person after confirming the number of healthcare work groups that could participate in the study. After excluding incomplete questionnaires, this study collected 33 groups and 214 group members from two hospitals, including 11 groups in the medical center at Northern Taiwan, all of which are groups of nurses, and 22 groups in the regional teaching hospital at Central Taiwan. The average group size is 11.7 people. Among the 33 groups, there are 24 groups of nurses (72.7%), accounting for the majority, followed by six administrative groups (18.2%); the other groups are one pharmacist group, one medical technologist group, and one physical therapist group. Each group unit leader is the leader of the group. Of the group leaders, 30 are women (90.9%), accounting for the vast majority, and only three are male. Ten leaders (30.3%) had the job tenure for under 5 years, nine (27.2%) for 6–10 years, five (15.2%) for 11–15 years, five (15.2%) for 16–20 years and four (12.1%) for over 21 years. In terms of the educational level, 16 (48.5%) were below junior university, and 17 (51.5%) were university and above. Eleven (33.4%) were younger than 30 years old, eight (24.2%) were 31–35 years old, seven (21.2%) were 36–40 years old, and seven (21.2%) were 41 years old or older. There were 20 unmarried persons (60.6%) and 13 married persons (39.4%). According to the analysis of the demographic background of the group members, 125 (58.4%) were under 5 years of job tenure, 53 (24.8%) had 6–10 years of service, 18 (8.4%) had 11–15 years of service, 15 (7%) had 16–20 years of service, and three (1.4%) were in the service over 21 years. In terms of educational level, 88 (41.1%) were below junior university and 126 (58.9%) were the university and above. A total of 149 (69.6%) were under 30 years, 37 (17.2%) were between 31–35 years old, 22 (10.2%) were between 36–40 years old, and six (3%) were over 41 years. There were 161 unmarried persons (75.2%), 52 married persons (24.2%), and one other (0.6%). 

### 2.2. Measurements

A seven-point scale was used for all of the measures. The response options are from 1 = “strongly disagree” to 7 = “strongly agree.” Since the survey was conducted in Taiwan, the method of back translation was used in this study to make sure all the scales have been translated from English into Chinese properly. The questionnaires for a work group were divided into two categories: there was a questionnaire for the group leader, and there were several questionnaires for group members. The one for group leaders covered the basic information of the group and transformational leadership. The group leaders were asked to rate their style of transformational leadership and provide some information for the work groups. Questionnaires for group members involved the three key variables at the individual level. Group members were asked to rate the aspects of work engagement, intrinsic motivation, knowledge sharing, and control variables. The questionnaires were reviewed and approved by the Institutional Review Board of the hospital (HP150030_CCGH IRB) for implementation.

Knowledge sharing. The scale developed by Van de Hooff and De Ridder (2004) [47] was adopted to measure employees’ knowledge sharing; it includes 10 questions related to knowledge sharing. There are two dimensions in this construct, namely knowledge donation and collection. Group members were responsible to evaluate their performance of knowledge sharing. Sample items like “I share my skills with colleagues within my work group.” and “Colleagues within my work group tell me what they know when I ask them about it.” The Cronbach’s α for this scale was 0.93. 

Intrinsic motivation. Intrinsic motivation in this study mainly measures employees’ intrinsic motivation toward knowledge sharing. This study adopted the items developed by Foss et al. (2009) [14]. There were three measurement items in total, which were addressed by the group members to evaluate their own intrinsic motivation, such as: why do you share knowledge with others? “I think it is an important part of my job.” The Cronbach’s α for this scale was 0.92.

Work engagement. This study adopted the short version scale by Schaufeli et al. (2006) [71]. This construct includes three dimensions, which are vigor, dedication and absorption. Sample items like: “At my work, I feel bursting with energy”, and “I get carried away when I am working”, were provided to the members to evaluate their work engagement. The Cronbach’s α for this scale was 0.95. 

Transformational leadership. The scale developed by Bass and Avolio (1992) [72] was adapted to measure transformational leadership, and included 12 items. There are four dimensions under this construct. These are idealized influence, inspirational motivation, intellectual stimulation and individualized consideration. This scale was offered to the group leaders to evaluate their transformational leadership. Sample items like “I make others feel good to be around me.” The Cronbach’s α for this scale was 0.94.

Control variables. At the individual level, this study used member demographic variables such as age, education and working tenure, as the control variables. We measured group members’ age, education (measured as six levels: elementary school or below, junior high school, senior high school, associate’s degree, bachelor’s degree, master’s degree and PhD) and number of working years. At the group level, this study also used group size and group leader demographic variables like age, education and working tenure as control variables. In addition, previous studies suggested that mutual trust among group members would prompt knowledge sharing [15]. Therefore, this study also used group trust as a control variable. Three measurement items used by Wu and Lee (2016) [15] were adopted for members to evaluate the trust among group members. The Cronbach’s α for this scale was 0.82. Since group trust is a construct at the group level, we further tested the within-group agreement for group trust by computing the intraclass correlation coefficient (ICC1) and within-group inter-rater agreement (rwg). The ICC1 was 15.58%, while the mean value of rwg was 0.90 and the lowest value was 0.73. As a result, the values of rwg and ICC1 are well above acceptable levels [73]. Therefore, the aggregated measure of group trust is justified.

### 2.3. Measurement Validation

This study also employed a four-factor confirmatory factor analysis model for the above four measures at the individual level (i.e., knowledge sharing, intrinsic motivation, work engagement and perception of group trust). In order to keep a reasonable number of degrees of freedom, item parceling was used in the model [74]. This model achieved an acceptable fit: GFI = 0.95, AGFI = 0.90, CFI = 0.98 and RMSEA = 0.059. All of the measures had a composite reliability (CR) above 0.78 and average variance extracted (AVE) above 0.61. The square roots of all AVE scores were higher than any correlations of possible focal pair measures. Therefore, both convergent validity and discriminant validity were supported. Moreover, group members conducted measurements regarding the main variables that might result in a potential problem, namely common method bias. To reduce the negative effect of this problem, this study asked participants to complete the dependent variable questions before the others. In addition, Harman’s one-factor test [75] also showed no serious problem regarding common method bias. 

## 3. Results

This study includes the analysis of both individual and group levels. Thus, the hierarchical linear modeling (HLM) analysis technique was applied in this study. Having a significant between-group variance in the dependent variables of interest is a basic requirement in conducting HLM models. There are two dependent variables in this study: knowledge sharing and intrinsic motivation. This study first estimated a null model for each of these two variables. The results showed that knowledge sharing (τ00 = 0.14, *p* < 0.001; ICC = 0.16) and intrinsic motivation (τ00 = 0.26, *p* < 0.001; ICC = 0.20) have significant between-group variance. Thus, using an HLM analysis is justified by the data. Table 1 provides the means, standard deviations, and correlations of the variables used in this study. As shown in Table 1, work engagement is positively correlated with knowledge sharing (r = 0.49, *p* < 0.001) and intrinsic motivation (r = 0.63, *p* < 0.001), these results provide some initial evidence for hypothesis 1 and 2, respectively. Meanwhile, intrinsic motivation is also positively correlated with knowledge sharing (r = 0.59, *p* < 0.001); combined with the positive correlation between work engagement and intrinsic motivation, this implies that intrinsic motivation might be a mediator in the relationship between work engagement and knowledge sharing. Thus, we also have some evidence for Hypothesis 3. 

Table 2 summarizes the results of testing Hypotheses 1, 2, 3 and 4 from the HLM analyses. Hypothesis 1 argues that work engagement has a positive influence on knowledge sharing. Model 1 of Table 2 demonstrates that work engagement is positively and significantly related to knowledge sharing (M1, γ = 0.31, *p* < 0.001); thus, Hypothesis 1 is supported. Hypothesis 2 argues that work engagement has a positive influence on intrinsic motivation. Model 4 of Table 2 shows that work engagement is positively and significantly related to intrinsic motivation (M4, γ = 0.52, *p* < 0.001), thus supporting Hypothesis 2. Hypothesis 3 proposes that intrinsic motivation would mediate the relationship between work engagement and knowledge sharing. This study took the approach suggested by Kenny et al. (1998) [76] to test this mediating effect. The results in Model 2 of Table 2 indicate that when we tested the influences of work engagement and intrinsic motivation together, intrinsic motivation had a significant impact on knowledge sharing (M2, γ = 0.39, *p* < 0.001); however, the influence of work engagement is non-significant (M2, γ = 0.09, n.s.). Therefore, intrinsic motivation fully mediates the relationship between work engagement and knowledge sharing, and Hypothesis 3 is supported.

**Figure 2:**
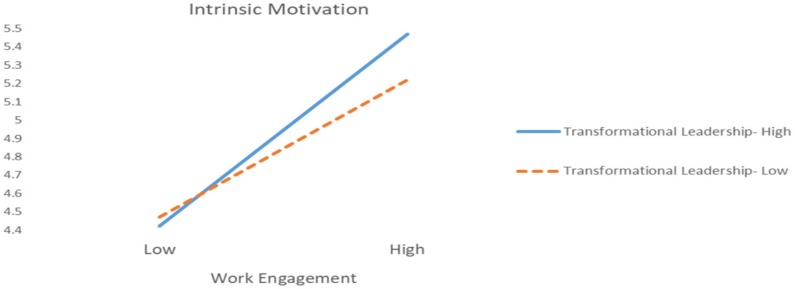
Plot of interaction between work engagement and transformational leadership on intrinsic motivation.

Hypothesis 4 proposes that transformational leadership has a positive impact on knowledge sharing. The results in Model 3 of Table 2 indicate that transformational leadership does not have a significant effect on knowledge sharing (M3, γ = 0.09, n.s.). Therefore, Hypothesis 4 is not supported. Hypothesis 5 proposes that transformational leadership would strengthen the relationship between work engagement and intrinsic motivation. Model 6 of Table 3 indicates that the interaction term of transformational leadership and work engagement is positively and significantly related to intrinsic motivation (M6, γ = 0.17, *p* < 0.01). In addition, this study also graphs the interaction effect in Figure 2. In Figure 2, we can clearly see that the slope of the relationship between work engagement and intrinsic motivation is stronger under the condition of high transformational leadership than under the condition of low transformational leadership; thus, Hypothesis 5 is supported.

Hypothesis 6 proposes that high transformational leadership would strengthen an indirect effect of intrinsic motivation between work engagement and knowledge sharing. In order to test this hypothesized moderated mediation effect, this study followed the suggestion of Muller, Judd and Yzerbyt (2005) [77] and examined three conditions accordingly: (1) a significant effect of work engagement on knowledge sharing, (2) a significant interaction between work engagement and transformational leadership on intrinsic motivation, and (3) a signification effect of intrinsic motivation on knowledge sharing. The relevant three-step analysis is shown in Table 3. As shown in Model 5 of Table 3, work engagement is positively and significantly related to knowledge sharing (M5, γ = 0.31, *p* < 0.001), supporting Condition 1. Next, the result of testing for Hypothesis 5 satisfied the second condition: that the interaction term of transformational leadership and work engagement has an impact on intrinsic motivation, as shown in Model 6. Finally, Model 7 reveals that intrinsic motivation is positively and significantly related to knowledge sharing (M7, γ = 0.40, *p* < 0.001), lending support to condition 3. As a result, the three conditions are satisfied and Hypothesis 6 is supported.

This study also used Hayes’ (2013) PROCESS to estimate this conditional indirect effect and obtain bias-corrected bootstrapped confidence intervals (using 1000 bootstrap samples) [78]. The result of PROCESS shows that the difference between indirect effects (work engagement on knowledge sharing via intrinsic motivation) at the different values of transformational leadership is significantly varied, with a 95% CI of [0.0011, 0.1046], not including zero. Table 4 also shows that the indirect effect at a high level of transformational leadership (0.2669) is stronger than the indirect effect at a low level of transformational leadership (0.1859). In other words, either the result from the method of Muller et al. (2005) [77] or PROCESS supports Hypothesis 6.

## 4. Discussion

Drawing from the COR theory and insights from STD, this study contributes to the knowledge-sharing literature by highlighting that knowledge sharing could be one kind of active behavior when individuals possess abundant resources. For the personal resources, this study finds that both work engagement and intrinsic motivation influence knowledge sharing. In addition, intrinsic motivation plays a mediating role between work engagement and knowledge sharing. For the external resources, the results show that transformational leadership strengthens the relationship between work engagement and intrinsic motivation. Moreover, the indirect effect of work engagement on knowledge sharing through intrinsic motivation is conditional upon the level of transformational leadership.

The results of this study can contribute to the knowledge-sharing literature in several ways. First, this study examined the indirect effect of work engagement on knowledge sharing through intrinsic motivation. Based on the concept of the gain spiral of resources from the COR theory [29,30], this study proves that engaged employees can generate more intrinsic motivation and then achieve a higher level of knowledge sharing. In other words, employees’ personal resources can cause a positive spiral of gaining resources and then promote knowledge sharing. This study is the first to provide initial evidence that the concept of the gain spiral of resources might be a useful perspective by which to explore the mechanism between personal resources and knowledge sharing. Although previous studies have applied the COR theory into knowledge-sharing studies [15,16], they focus on the cycle of loss rather than the cycle of gain. In this study, we highlight the importance of the gain spiral, in that individuals with abundant resources would have more resources and opportunities to use their current resources to achieve a higher level of knowledge sharing. 

Second, the results of this study remind us that there should be more research examining the positive side of individuals’ resources in regard to the issue of knowledge sharing. This research direction also responds to the call from positive organizational behavior [79,80,81] that scholars should put more attention on the positive side of employees. Thus, future studies could further explore the relationship between positive resources of individuals and knowledge sharing. In other words, future studies can use some other important personal resources besides work engagement as the main predictors of knowledge sharing. For instance, flourishing is an important and emergent concept in recent years. Flourishing employees are in a good state of well-being and have abundant resources [33]; they might be more willing to engage in knowledge sharing.

Finally, regarding the role of transformational leadership, we do not find a direct influence on knowledge sharing. One potential reason might be that group trust already accounts for most of the explanation for the direct influences from the group level. However, this study clearly shows that transformational leadership can play an important role as a facilitator of the relationship between work engagement and intrinsic motivation. This finding is consistent with the argument of the COR theory that an individual’s different kinds of resources could aggregate and become a resource pool which would help the individual [29,30]. In spite of transformational leadership not having a direct influence on knowledge sharing, it has a significant moderating effect. This shows that positive leadership could be an important facilitator of knowledge sharing. When led by leaders with high transformational leadership, members’ work engagement is more likely to develop intrinsic motivation that, in turn, promotes knowledge sharing. Future studies can further explore whether other types of positive leadership, such as empowering leadership [82] or ethical leadership [83], could also become a facilitator of knowledge sharing. Moreover, according to the COR theory [29,30], gaining positive resources could help individuals to deal with unfavorable challenges. Future studies could further explore whether transformational leadership could also act as a buffer for employees when they face harmful situations related to knowledge sharing. 

The findings of this study also have important practical implications. First, employees’ work engagement could have a positive series effect on knowledge sharing. Given the positive influence of work engagement, organizations should help to increase employees’ level of work engagement by providing more job-related resources. For example, organizations can establish well-designed social support, performance feedback, and skill variety for employees to increase their job resources and then increase work engagement. Moreover, this study also shows that transformational leadership can enhance the positive effect of work engagement. Organizations should pay more attention when they select leaders for the work groups. Some of the characteristics of transformational leadership could become important criteria in the selection. Likewise, organizations could provide leadership-training programs for current leaders in order to develop their transformational leadership skills. 

Basically, patient-centered high-quality services are the mission of professional healthcare workers in the context of health care. As the complexity of medical care increases, the professional level of healthcare workers needs to keep pace with the times, so the knowledge sharing among healthcare workers seems more and more important. The results of this study show that, as healthcare workers become more enthusiastic about their work, their intrinsic motivation for knowledge sharing will be improved, thus showing more knowledge sharing behaviors. A higher degree of knowledge sharing among members will help medical institutions to further optimize the quality of medical care and drive group members to become innovative groups. In particular, when the head of a medical unit leads his or her subordinates in a transformational leadership manner, it can further strengthen the engaged healthcare workers to demonstrate knowledge-sharing behavior. When members of the healthcare group are generally willing to engage in knowledge sharing, the quality and performance of medical care in the entire medical institution can be further improved.

### Limitations

There are some limitations in this study. First, since the measurement of knowledge sharing is rated by employee self-reporting, it might be overestimated. Future studies could measure this scale by the employees’ colleagues or supervisors. Second, although the hypotheses in this study imply causal relationships, all of the empirical data were collected at the same time. Future studies might consider collecting data with a longitudinal design. Third, this study only uses the sample of healthcare workers. Future studies might collect different types of samples to increase the generalization of the findings. Finally, we consider that an individual’s personal and external resources have a conjoint influence, such as a moderating effect. However, this study overlooks the fact that personal resource and external resource might have a causal relation. For example, a positive leadership might enhance employees’ personal resources. Future studies can further explore the causal relationship between external resources and personal resources while they discuss the relationship between employees’ resources and knowledge sharing.

## 5. Conclusions

Based on the COR theory, this study proposes and tests a multilevel theoretical model of relationships among work engagement, intrinsic motivation, transformational leadership, and knowledge sharing. Work engagement and intrinsic motivation are considered as employees’ personal resources, and transformational leadership is thought of as employees’ external resourced. The results of this study show that work engagement has an indirect effect on knowledge sharing through intrinsic motivation. Moreover, transformational leadership positively moderates the relationship between work engagement and intrinsic motivation, and then strengthens the indirect effect of work engagement on knowledge sharing. According to the findings of this study, it can be concluded that employees’ current resources in the workplace are important determinants for knowledge sharing. This is a pioneer study to discuss the relationship between employees’ positive resources and knowledge sharing. Drawing from the COR theory, future research could explore the relationship between employee’s different kinds of positive resources and knowledge sharing.

## Figures and Tables

**Table 1:** Means, standard deviations, and correlations.

Individual Level Variables	Mean	s.d.	1	2	3	4	5	6
1. Age	27.74	6.91						
2. Education	4.57	0.55	0.23 **					
3. Working tenure	6.11	5.49	0.76 ***	−0.14 *				
4. Work engagement	4.11	1.20	−0.09	−0.14 *	0.07	(0.95)		
5. Intrinsic motivation	4.86	1.14	−0.19 **	−0.05	−0.06	0.63 ***	(0.92)	
6. Knowledge sharing	4.95	0.93	−0.15 *	−0.02	−0.10	0.49 ***	0.59 ***	(0.93)
Group level variables	Mean	s.d.	1	2	3	4	5	6
1. Group size	11.70	7.42						
2. Leader age	35.19	8.45	−0.08					
3. Leader education	4.58	0.71	0.19	−0.09				
4. Leader tenure	10.76	7.20	0.09	0.83 ***	−0.24			
5. Group trust	5.23	0.46	−0.37 *	0.24	−0.04	0.01	(0.82)	
6. Transformational leadership	4.83	0.81	0.01	0.21	0.13	0.20	0.20	(0.94)

Reliabilities are on the diagonal parentheses. * *p* < 0.05, ** *p* < 0.01, *** *p* < 0.001. Sample size for the group level is 33, and sample size for the individual level is 214.

**Table 2:** Results of hierarchical linear modeling (HLM) analyses.

Variable	Knowledge Sharing			Intrinsic Motivation
	Model 1	Model 2	Model 3	Model 4
Level 1				
Age	0.01 ^a^	0.01	0.01	−0.03
Education	0.02	−0.02	−0.03	0.18
Tenure	−0.03	−0.03	−0.03	0.00
Work engagement	0.31 ***	0.09	0.09	0.52 ***
Intrinsic motivation		0.39 ***	0.39 ***	
Level 2				
Group size	0.00	0.00	0.00	0.01
Leader age	0.00	0.00	0.00	0.01
Leader education	0.10	0.16	0.14	−0.01
Leader tenure	0.01	0.00	0.00	0.00
Group trust	0.45 ***	0.29 **	0.24**	0.44 *
Transformational leadership			0.09	
Within-group residual variance	0.49	0.39	0.39	0.63
△R^2^_within-group_^b^	31.90%	45.97%	45.90%	40.04%
Deviance	536.76	502.02	505.84	577.10

^a^ Not standardized coefficients in HLM results. ^b^ Difference compared to the null Model. * *p* < 0.05 ** *p* < 0.01 *** *p* < 0.001.

**Table 3:** Results for testing mediated moderation by transformational leadership.

Variable	Knowledge Sharing	Intrinsic Motivation	Knowledge Sharing
	Step 1	Step 2	Step 3
	Model 5	Model 6	Model 7
Level 1			
Age	0.01 ^a^	−0.02	0.01
Education	0.02	0.18	−0.03
Tenure	−0.03	0.00	−0.03
Work engagement (WE)	0.31 ***	0.50 ***	0.10
Transformational leadership X WE	0.01	0.17**	−0.09
Intrinsic motivation (IM)			0.40 ***
Transformational leadership X WE			0.05
Level 2			
Group size	0.00	0.01	0.00
Leader age	0.00	0.01	0.00
Leader education	0.09	0.00	0.15
Leader tenure	0.00	0.01	0.00
Group trust	0.42 ***	0.53 **	0.21 *
Transformational leadership	0.06	−0.03	0.07
Within-group residual variance	0.49	0.64	0.39
△R^2^_within-group_^b^	31.36%	39.14%	45.26%
Deviance	543.14	577.98	511.69

^a^ Not standardized coefficients in HLM results. ^b^ Difference compared to the null Model. * *p* < 0.05 ** *p* < 0.01 *** *p* < 0.001.

**Table 4:** Moderated mediation test of PROCESS.

Moderator	Level	Conditional Indirect Effect	SE	LL 95% CI	UL 95% CI
**Transformational leadership**	Low (−1sd)	0.1859	0.0600	0.0851	0.3071
	High (+1sd)	0.2669	0.0505	0.1738	0.3708

Note. Bootstrap sample size = 1000. CI = confidence interval; LL = lower limit; UL = upper limit.
